# Mental disorders in the peripartum period: A review

**DOI:** 10.1192/j.eurpsy.2025.2423

**Published:** 2025-08-26

**Authors:** A. M. Morales Rivero, F. A. Rodríguez Batista, E. E. Morales Castellano, P. Rivero Rodríguez, M. Martínez Grimal

**Affiliations:** 1 Hospital Universitario de Gran Canaria Doctor Negrín, Las Palmas de Gran Canaria, Spain

## Abstract

**Introduction:**

Perinatal mental illness refers to all psychiatric disorders that occur during pregnancy and up to one year after childbirth. For a long time, pregnancy was believed to be a protective factor for women, representing a period of emotional well-being. However, research has shown that pregnancy does not shield women from the onset or persistence of psychiatric disorders. Instead, it poses a risk factor. The mother’s mental state directly influences the cognitive, emotional, behavioral, and physical development of the child. Mothers experiencing mental health issues during this critical period may struggle with bonding with the baby, and these difficulties might persist even after the resolution of the triggering mental disorder. Hence, early diagnosis and treatment are crucial.

**Objectives:**

The objective of this study is to review the available evidence on mental disorders in the peripartum period, focusing on the most common ones (postpartum blues, postpartum depression, and postpartum psychosis). Additionally, we will discuss data regarding the most severe consequences, such as maternal suicide and filicide.

**Methods:**

A systematic literature review was conducted, analyzing documents published in various scientific journals with online publications.

**Results:**

The prevalence is high; depending on the studies, between 15-20% of women experience some form of treatable pathology during pregnancy and postpartum. We will discuss postpartum blues, postpartum depression, and postpartum psychosis. Postpartum blues are characterized by transient mood disturbances (spontaneous relief within 2-3 weeks) occurring between the first day and the third week postpartum. Symptoms include emotional lability, episodes of crying, sadness, anxiety, irritability, fatigue, and mild depressive mood. Postpartum depression is a clinical syndrome characterized by moderate to severe depressive symptoms lasting longer than postpartum blues and significantly impacting family life. It begins within the first 4 weeks after childbirth, although some studies date it between 6-7 weeks postpartum. Prevalence ranges from 5-15%. The term postpartum psychosis refers to an acute psychotic episode starting between 4-6 weeks after childbirth. Its prevalence in the general population is 0.1-0.2%, rising to 20-25% in individuals with personal or family history of affective disorders.

**Conclusions:**

Perinatal mental disorders are a public health problem with high prevalence, associated with complications in maternal health, pregnancy, neonatal outcomes, mother-infant bonding, and neonatal neurodevelopment. The perinatal period increases the risk of relapses in women with severe mental disorders and the onset of mental disorders.

**Disclosure of Interest:**

None Declared

